# Fosfomycin resistance in extended-spectrum beta-lactamase producing Escherichia coli isolated from urinary tract-infected patients in a tertiary care hospital

**DOI:** 10.1099/jmm.0.002039

**Published:** 2025-07-14

**Authors:** Priksha Thakur, Narinder Kaur, Shubham Chauhan, Reham Abdelmonem, Richard Donkor Amponsah

**Affiliations:** 1 Department of Microbiology, Maharishi Markandeshwar Institute of Medical Science and Research, Maharishi Markandeshwar (Deemed to be) University, Mullana, Haryana, India; 2 Lancashire Teaching Hospitals NHS Foundation Trust, Lancashire, UK; 3 Department of Medical Microbiology, University of Ghana Medical School, Accra, Ghana

**Keywords:** *Escherichia coli*, extended-spectrum beta-lactamases, fosfomycin resistance, urinary tract infections

## Abstract

*A corrigendum of this article has been published full details can be found at* *https://doi.org/10.1099/jmm.0.002076*

**Introduction.** Urinary tract infections (UTIs) are a significant global health concern, with *Escherichia coli* being the predominant pathogen responsible for uncomplicated and complicated cases. Fosfomycin has emerged as a promising oral treatment option for multidrug-resistant UTIs, particularly those caused by extended-spectrum *β*-lactamase (ESBL)-producing *E. coli*. However, fosfomycin resistance has been paralleled by its irrational use and the emergence of enzymes that modify fosfomycin in ESBL-producing *Enterobacteriaceae*, especially in Asia.

**Hypothesis/Gap Statement.** There is limited data on the prevalence of fosfomycin resistance among UTI patients in Northern Haryana, India. We hypothesize that demographic factors such as age, gender and patient type (inpatient vs. outpatient) may influence the prevalence of fosfomycin resistance and also provide insights into the effectiveness of fosfomycin in combating ESBL-producing *E. coli* infections in a tertiary care setting.

**Aim.** This study aimed to investigate the prevalence of fosfomycin resistance among ESBL-producing *E. coli* among UTI patients in a tertiary care hospital.

**Methodology.** Between March 2023 and February 2024, 7,348 urine samples were received from patients suspected of UTIs. The samples were subjected to screening using wet film examination and standard microbiological methods. Antibiotic susceptibility testing was done by VITEK-2 Compact (using an N-235 card), and ESBL production was confirmed using the combination disc diffusion test.

**Results.** Out of 7,348 urine samples, 1,176 (16%) were culture-positive, with *E. coli* accounting for 57% of the isolates. Among the 385 *E. coli* isolates, 224 (58%) were ESBL producers. Fosfomycin demonstrated high efficacy, with 95% susceptibility among ESBL-producing *E. coli* and 96% among non-ESBL producers. However, 5% of ESBL-producing *E. coli* isolates were resistant to fosfomycin. Resistance to other antibiotics, such as nalidixic acid (98%) and ampicillin (93%), was notably high. No significant associations were found between ESBL production and demographic factors such as age, gender or patient type (outpatient vs. inpatient).

**Conclusion.** Fosfomycin remains a highly effective treatment option for ESBL-producing *E. coli* UTIs in Northern Haryana, India, with low resistance rates observed. However, the emergence of fosfomycin resistance, albeit minimal, highlights the need for continuous surveillance and rational use of antibiotics to combat the growing threat of antimicrobial resistance.

## Introduction

Urinary tract infections (UTIs) are one of the most common causes of morbidity globally [1]. UTI is common in all age groups and is caused by various pathogens [1,2]. It is estimated that about 12% of men and children and 50% of all women experience UTI in their lifetime [3,5]. Clinical manifestations of UTI include pyelonephritis, asymptomatic bacteriuria and chronic or recurring infections, in addition to cystitis which is the most common presentation of UTI [6]. If the bacteria go up the urinary tract, they can cause pyelonephritis if not treated properly [7]. UTIs are classified into two main types: uncomplicated and complicated. Uncomplicated UTIs usually occur in healthy individuals who do not have problems with their urinary system [2,8, 9].

On the other hand, complicated UTIs occur in people with conditions that make them vulnerable, such as blockages in the urinary tract, nerve damage that leads to urine retention, weakened immune systems, kidney problems, pregnancy or the presence of medical devices like catheters or kidney stones [10,11]. Gram-negative and Gram-positive bacteria and certain fungi are associated with UTI [2]. *Escherichia coli* is the predominant pathogen causing UTIs, with increasing antibiotic resistance posing a significant challenge. Studies have reported high resistance rates to commonly prescribed antibiotics, including ciprofloxacin, amoxicillin and trimethoprim/sulfamethoxazole [12,14]. The prevalence of multidrug-resistant (MDR) and extended-spectrum *β*-lactamase (ESBL)-producing strains has also been on the rise [1], with fosfomycin emerging as a promising oral treatment option for these infections.

Studies have shown high susceptibility of ESBL-producing *E. coli* to fosfomycin, with Patwardhan *et al*. [15] reporting a 91.67% susceptibility rate. However, fosfomycin resistance has been observed to rise in parallel with increased usage, suggesting that selective pressure may be driving resistance [16]. Also, ESBL-producing *Enterobacteriaceae* are frequently exposed to broad-spectrum antibiotics, which may co-select for resistance to fosfomycin [17]. Several studies in Asia [18,20] have reported that fosfomycin-modifying enzymes, such as fosA genes, are often located on plasmids that also harbour ESBL or carbapenemase genes, enabling horizontal gene transfer and resistance. This genetic linkage increases the likelihood of fosfomycin resistance in ESBL-producing strains isolated from Asia. Additionally, biofilm-forming potential commonly seen in ESBL-producing *E. coli* [21,22] may further contribute to reduced susceptibility to fosfomycin by limiting drug penetration and promoting persistence of resistant strains. Furthermore, a prevalence study of pathogens among UTI patients in Northern India reported on the need for a community awareness programme for adherence to treatment protocol, considering emerging MDR strains, variability in pathogen prevalence in tertiary care settings and the need for category-specific (inpatient vs. outpatient) antibiotic policies [23].

As such, we hypothesized that the prevalence of fosfomycin resistance in ESBL-producing *E. coli* would be significantly higher in our study population in the Northern region of Haryana, India. Given *E. coli*’s dominance in UTIs and its high ESBL prevalence [2], we focused on this pathogen to optimize resource allocation for resistance surveillance. In addition, demographic factors such as age, gender and patient type (inpatient vs. outpatient) might impact the prevalence of fosfomycin resistance among ESBL-producing *E. coli*. To our knowledge, this is the first study to examine the prevalence of fosfomycin resistance among ESBL-producing *E. coli* from a tertiary care hospital in Northern India and also determine the frequency of resistance to fosfomycin in both inpatient and outpatient groups over 1 year.

## Methods

In this cross-sectional study, urine samples from outpatients and inpatients presenting with symptoms indicative of urinary tract infection, such as dysuria, increased and urgent urination and cloudy or bloody urine, were sent to the microbiology laboratory at the Maharishi Markandeshwar Institute of Medical Science and Research, Mullana, India, from March 2023 to February 2024. Only symptomatic patients for whom the attending clinician had requested a urine culture were included in the study. The study included 7,348 urine samples and comprehensive documentation of patient clinical data.

### Sample collection

Mid-stream urine was collected in sterile universal containers, with standard protocol. Subsequently, urine samples were transported to the laboratory without delay. If there were delays for more than 1–2 h, samples were stored in a refrigerator at 4 °C or transported in a refrigerated container. Urine samples received from suspected patients with UTI were included. Subsequently, only urine samples that were culture-positive for *E. coli* isolates were included in the study.

### Laboratory analysis

Urine was microscopically examined through wet film preparation to detect the presence of increased pus cells (>5 white blood cells per high power field) [24,25], which is an indication of urinary tract infection. The samples were then routinely inoculated on cystine–lactose–electrolyte-deficient agar and aerobically incubated at 37 °C for 18–24 h. The growth of organisms and bacteria count was done per Kass Phenomena [26]. Isolates were identified by standard biochemical tests.

### Antibiotic susceptibility testing

Antibiotic susceptibility testing was performed using the VITEK-2 Compact system (bioMérieux, France) with the N-235 card, following the manufacturer’s guidelines. The tested antibiotics included ampicillin, amoxicillin/clavulanic acid, ticarcillin, piperacillin/tazobactam, cefalotin, cefoxitin, cefixime, ceftazidime, ceftriaxone, ertapenem, amikacin, gentamicin, nalidixic acid, ciprofloxacin, norfloxacin, ofloxacin, fosfomycin, nitrofurantoin and trimethoprim/sulfamethoxazole. Interpretation of results adhered to the performance standards for antimicrobial susceptibility testing for all antibiotics provided by the Clinical and Laboratory Standards Institute (CLSI) [27].

### ESBL confirmation (combination disc diffusion test)

ESBL production was confirmed using the combination disc diffusion test [28,29]. A 0.5 McFarland suspension of the isolate was lawn-cultured on Mueller–Hinton agar. Discs of ceftazidime (30 µg) and ceftazidime/clavulanic acid (30/10 µg) were placed 20 mm apart. Plates were incubated at 37 °C for 18–24 h. ESBL positivity was defined as a ≥5 mm increase in the zone diameter of ceftazidime/clavulanic acid compared to ceftazidime alone.

### Data analysis

All statistical analyses were conducted using IBM SPSS Statistics 27 (version 27.0.1). Descriptive statistics were used to summarize demographic characteristics, including age, gender and patient type. Categorical variables were presented as frequencies and percentages. The Pearson chi-square (*χ*²) test was used to assess whether significant differences existed in ESBL positivity across different age groups, genders and patient types. To account for multiple comparisons among demographic variables (age, gender and patient type), a Bonferroni correction was applied, adjusting the significance threshold to 0.017 (0.05/3). For categorical variables with a 2×2 contingency table, Fisher’s exact test was reported when expected cell counts were below 5.

## Results

Out of the 7,348 urine samples, 1,176 (16%) were culture-positive and 6,172 (84%) were sterile. *E. coli* was 57% of the total culture-positive urine samples, with most (43%) isolated from people aged 19–45 and fewer isolated from younger groups (0–18 years). Also, slightly more women (54%) than men (46%) were infected. Moreover, *E. coli* was more frequently isolated from outpatients than inpatients (Table 1). Based on the distribution of ESBL among patients (Table 2) more men had positive cases than women. Also, ESBL-positive cases were prevalent among ages 19 to 45.

**Table 1.** Distribution of *E. coli* isolates (*n*=385)

Characteristic	No. of *E. coli* isolate	Percentage (%)
**Age**		
0–18	21	5
19–45	165	43
46–60	86	22
>60	113	30
**Gender**		
Male	180	46
Female	205	54
**Patient type**		
OPD	197	51
IPD	188	49

**Table 2.** Distribution of ESBL-positive and ESBL-negative cases across demographics

Characteristic	ESBL-positive (*N*)	ESBL-negative (*N*)	*P*-value
**Age**			0.457
0–18	10	11	
19–45	92	74	
46–60	51	34	
>60	71	42	
**Gender**			0.087
Male	113	67	
Female	111	94	
**Patient type**			0.115
OPD	107	90	
IPD	117	71	

*P*<0.05 indicates a significant relationship.

*P*≥0.05 indicates no significant relationship.

N, number of isolates.

### Antibiotic susceptibility pattern of *E. coli*

The highest resistance was seen for alidixic acid (98%), followed by ampicillin (93%) and cefalotin (91%). On the other hand, high sensitivity was recorded for ertapenem (98%), followed by fosfomycin (95%) (Table 3).

**Table 3.** The antimicrobial susceptibility pattern of *E. coli* isolates (*n=*385)

Antibiotic	Resistance % (*N*)	Sensitive % (*N*)	Intermediate % (*N*)
Ampicillin	93 (332)	5 (16)	3 (9)
Amoxicillin/clavulanic acid	58 (221)	27 (101)	15 (55)
Ticarcillin	91 (340)	9 (32)	n/a
Piperacillin/tazobactam	55 (203)	45 (169)	n/a
Cefalotin	92 (331)	8 (30)	n/a
Cefoxitin	55 (201)	40 (147)	5 (15)
Cefixime	89 (316)	10 (35)	1 (1)
Ceftazidime	70 (256)	28 (106)	2 (4)
Ceftriaxone	88 (333)	12 (47)	n/a
Ertapenem	2 (6)	98 (248)	n/a
Amikacin	31 (120)	62 (239)	7 (26)
Gentamicin	42 (161)	58 (224)	n/a
Nalidixic acid	98 (356)	2 (5)	n/a
Ciprofloxacin	83 (320)	10 (38)	7 (28)
Norfloxacin	79 (301)	20 (80)	1 (2)
Ofloxacin	81 (308)	19 (73)	n/a
Fosfomycin	5 (18)	95 (367)	n/a
Nitrofurantoin	12 (46)	80 (298)	8 (26)
Trimethoprim/sulfamethoxazole	63 (244)	37 (141)	n/a

*N*, number of isolates; n/a, not applicable.

### Antibiotic resistance patterns in ESBL and non-ESBL-producing *E. coli*

Based on the confirmatory combination disc diffusion test, 224 (58%) of *E. coli* isolates were ESBL producers, while 161 (42%) strains were non-ESBL producers. The highest resistance was seen in nalidixic acid (99%) against ESBL-producing *E. coli*, followed by ampicillin (98%), cefalotin (98%), cefixime (98%) and ticarcillin (97%) (Table 4). Out of the 224 ESBL-producing *E. coli*, 11 (5%) were fosfomycin-resistant strains. Also, 7 out of the 161 non-ESBL-producing *E. coli* strains were resistant to fosfomycin.

**Table 4.** The antibiotic resistance pattern of ESBL-producing *E. coli* isolates

Antibiotic	Resistance *N* (%)
**Nalidixic acid**	217 (99)
**Ampicillin**	212 (98)
**Cefalotin**	215 (98)
**Cefixime**	205 (98)
**Ticarcillin**	217 (97)
**Ceftriaxone**	212 (96)
**Ceftazidime**	206 (94)
**Ciprofloxacin**	199 (94)
**Amoxicillin/clavulanic acid**	155 (90)
**Ofloxacin**	194 (88)
**Norfloxacin**	193 (87)
**Piperacillin/tazobactam**	158 (72)
**Trimethoprim/sulfamethoxazole**	157 (70)
**Cefoxitin**	143 (68)
**Gentamicin**	118 (53)
**Amikacin**	90 (44)
**Nitrofurantoin**	37 (28)
**Fosfomycin**	11 (5)
**Ertapenem**	5 (4)

*N*, number of isolates.

## Discussion

Although we benefit greatly from antibiotic usage, overuse and misuse have contributed to the rise of resistance among uropathogenic bacteria, which is a serious public health threat. In UTI, the production of ESBL increases morbidity and mortality rates among infected patients [30]. Fosfomycin, which is the recommended treatment option against ESBL-producing *E. coli* (87%–99% susceptibility) [31], sometimes fails. This study was conducted to determine the prevalence of fosfomycin resistance in ESBL-producing *E. coli* isolated from urine samples in a tertiary hospital in India and also to find the frequency of resistance to fosfomycin in both inpatient and outpatient groups over 1 year. In this current study, a total of 7,348 urine samples were collected, of which 1,176 (16%) were culture-positive and 6,172 (84%) were sterile. A similar study by Magliano *et al*. [32] assessed 61,273 total urine samples, of which 13,820 (22%) were culture-positive, while 78% samples were sterile. Another study by Hasegan *et al*. [33] also analysed 15,389 urine samples, of which 1,530 (9.9%) were culture-positive, while the majority of the samples (89.1%) were sterile. In 2020, a northern Indian study that assessed the prevalence and antimicrobial sensitivity pattern of bacteria causing urinary tract infections had a culture positivity rate (17%) similar to that of our study (16%) [23].

Out of 1,176 culture-positive samples, *E. coli* (57%) was the predominant isolate, while 43% of the isolates were of other organisms. According to several studies, *E. coli* is the most predominant bacterium isolated from urine samples of UTI patients [34,37]. The majority of *E. coli* were isolated from individuals within the 19–45 age group, followed by the above 60 age group, the 46–60 age group and the 0–18 age group (Table 1). Similar findings were reported by Naseer *et al*. [38], who found that the majority of the *E. coli* (36%) were isolated from the 37–54 age group, followed by patients above 55 years and the 19–36 age group, respectively. A study by Magliano *et al*. [32] also found less prevalence of *E. coli* isolates in young age, particularly ages ≤14 years.

In this study, out of 385 *E. coli* isolates, 54% were from female patients and 46% from male patients. It has already been established that UTI exhibits sex bias, affecting more women than men, with women being 20–40 times more likely to have a UTI than men of the same age [39,41]. In a similar study, *E. coli* showed a higher percentage of UTI in females (55%) than in males (45%) [42]. Looking at the frequency of *E. coli* isolates between the two patient types, 51% were observed from outpatient department (OPD) patients as compared to 49% of inpatient department (IPD) patients. Similar to a study conducted in a tertiary care hospital in North West India, 47% of *E. coli* were isolated from IPD patients, while 53% were from OPD patients [43]. We examined the association between UTIs caused by ESBL-producing *E. coli* and age range, gender and patient type using chi-square tests. The results indicated no significant association between ESBL-producing *E. coli* and age range (*χ*²=2.603, *P*=0.457) or gender (*χ*²=2.935, *P*=0.087). Similarly, no significant association was found between ESBL status and patient (*χ*²=2.480, *P*=0.115). No significant association was observed between ESBL production and age group, gender or patient type after applying Bonferroni correction (adjusted *α*=0.017). These findings suggest that UTIs caused by ESBL-producing *E. coli* are not significantly influenced by age, gender or patient type (OPD vs. IPD) [44,45].

**Fig. 1.**
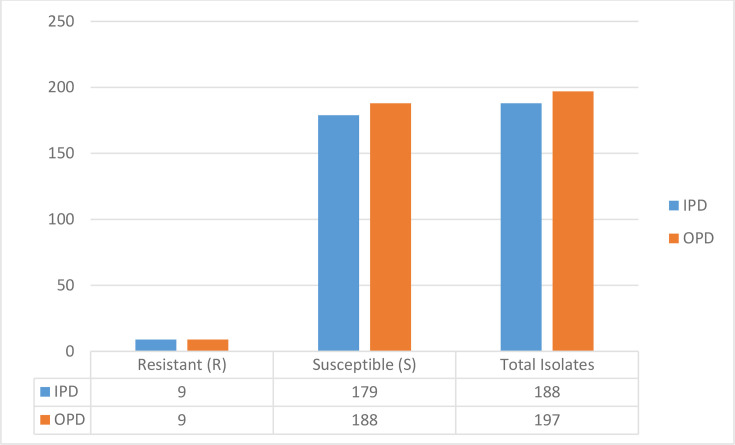
Fosfomycin resistance among *E. coli* isolated from inpatient and outpatient groups.

The highest resistance (98%) was observed for nalidixic acid against *E. coli*, followed by ampicillin (93%) and cefalothin (91%). High sensitivity was also observed for ertapenem (98%), followed by fosfomycin (95%). A systematic review and meta-analysis reported a high resistance of ampicillin (86%) and cefalothin (60%) to *E. coli* isolates in Iran [46]. The high rate of resistance in our study could be attributed to the small sample size and single-centre study design. In a comparative study, nalidixic acid was highly resistant (84%) to *E. coli* isolated from UTI patients [47]. Another study in Saudi Arabia reported a high sensitivity (99.2%) rate of ertapenem to *E. coli* isolates [48], similar to the 98% rate reported in our study. Lee *et al*. [49] observed the high susceptibility of fosfomycin (92.9%) in uropathogenic *E. coli*. Similarly, Wagle *et al*. [50] conducted a study on the susceptibility profile of fosfomycin to uropathogenic *E. coli* isolated at a Tertiary Care Hospital in Nepal and found 98% of isolates to be susceptible. Also, analysis of fosfomycin resistance among *E. coli* isolates revealed similar resistance rates in both inpatient and outpatient groups (Fig. 1).

Using the CLSI-recommended confirmatory combination disc diffusion test [28], 224 (58.1%) strains were ESBL producers. This is in concordance with studies that reported 58.1% [51], 58.5% [52] and 59% [53] ESBL-producing *E. coli* isolated from urine samples of UTI patients. While high resistance to *β*-lactam antibiotics is a characteristic of ESBL-producing *E. coli* due to their ability to hydrolyse penicillins and cephalosporins, understanding resistance to non-*β*-lactam agents is critical for guiding treatment decisions. In our study, ESBL-producing *E. coli* exhibited high resistance against quinolones, including nalidixic acid (99%), ciprofloxacin (94%), ofloxacin (88%) and norfloxacin (87%). A similar study conducted in India by Naik and Desai [54] showed high resistance for nalidixic acid 96.3% (79/82) and ciprofloxacin 87.8% (72/82) against ESBL-producing *E. coli*. Resistance to the folate pathway inhibitor trimethoprim/sulfamethoxazole was high (70%), limiting its clinical utility. This is in accordance with a study by Al-Mayahie and Al Kuriashy [55] where resistance was very high for trimethoprim/sulfamethoxazole (83.5%). Moderate resistance was seen for aminoglycosides such as gentamicin (53%) and amikacin (44%). Although amikacin has been previously suggested for use in outpatient parenteral antibiotic therapy programmes for ESBL-related UTIs [56], its moderate resistance rate in our study and the need for parenteral administration with close monitoring make it an impractical choice for managing mild to moderate UTIs in resource-limited settings. On the other hand, 95% of ESBL-producing *E. coli* isolates showed sensitivity towards fosfomycin, while only 5% were resistant. A study by Tseng *et al*. [57] also found the fosfomycin susceptibility rate for human ESBL-producing *E. coli* isolates to be 94%, while only 6% were resistant. Similarly, Mohamed *et al*. [58] reported high susceptibility of ESBL-producing *E. coli* to fosfomycin (99.03%) compared to its resistance (0.97%). Another study conducted by Ríos *et al*. [59] over 3 years found resistance rates to fosfomycin to be 4.3%, 5.45% and 6.6% for each year, respectively. Although the resistance rate of fosfomycin was low in our study, resistance towards fosfomycin should not be neglected because fosfomycin has shown effectiveness against ESBL-producing *E. coli* compared to other first-line antibiotics [58,60,62]. Of the total 161 non-ESBL-producing *E. coli* isolates, 96% showed sensitivity towards fosfomycin, while only 4% were resistant. In a similar study conducted by Ríos *et al*. [59], they observed that the rates of fosfomycin resistance in non-ESBL-producing isolates were 3.5%, 4.05% and 5.53% annually for 2013, 2018 and 2021, respectively. However, a study in Turkey reported a lower resistance rate (1.45%) of non-ESBL-producing *E. coli* to fosfomycin compared to our study [1].

## Conclusion

Fosfomycin demonstrated high efficacy against ESBL-producing *E. coli* isolates, reinforcing its role as a good treatment option for MDR UTIs. However, the emergence of fosfomycin resistance, albeit low, underscores the need for continuous surveillance and controlled use of antibiotics to combat the growing threat of antimicrobial resistance. These findings should be considered, and further studies should be conducted to evaluate fosfomycin resistance among ESBL-producing and non-ESBL-producing *E. coli* isolated from UTI patients.

## Abbreviations

CLSI: Clinical and Laboratory Standards Institute
ESBL: extended-spectrum beta-lactamase
IPD: inpatient department
MDR: multidrug-resistant
OPD: outpatient department
UTI: urinary tract infection
