# Corrigendum: Fosfomycin resistance in extended-spectrum beta-lactamase producing Escherichia coli isolated from urinary tract-infected patients in a tertiary care hospital

**DOI:** 10.1099/jmm.0.002076

**Published:** 2025-09-15

**Authors:** Priksha Thakur, Narinder Kaur, Shubham Chauhan, Reham Abdelmonem, Richard Donkor Amponsah

**Affiliations:** 1 Department of Microbiology, Maharishi Markandeshwar Institute of Medical Science and Research, Maharishi Markandeshwar (Deemed to be) University, Mullana, Haryana, India; 2 Lancashire Teaching Hospitals NHS Foundation Trust, Lancashire, UK; 3 Department of Medical Microbiology, University of Ghana Medical School, Accra, Ghana

In the original published version of the article, Figure 1 was included as a representative example of a wet mount of urine. The image used in Figure 1 did not portray original data or the experimental set-up from the research described within the article. The article did not make clear that this was a reference image by crediting the original source of the image, and the necessary permissions for reuse of the image were not obtained.

As the image is not essential to the scientific content of the article, the authors and the Publisher are removing Figure 1 from the published version of record of the article.

The authors acknowledge that the inclusion of this figure was misleading and apologise for any confusion caused.

